# Dual Mediating Effect of Electronic Device Utilization and Life Satisfaction on the Relationship between Instrumental Activities of Daily Living and Depression in Older Adults

**DOI:** 10.3390/ijerph191710617

**Published:** 2022-08-25

**Authors:** Hwa-Soo Koong, Mihye Lim, Kawoun Seo

**Affiliations:** 1 Department of Dental Hygiene, Konyang University, Daejeon 35365, Korea; 2 Department of Nursing, Joongbu University, Geumsan-gun 32713, Korea

**Keywords:** aged, instrumental activities of daily living, depression, electronic devices, life satisfaction

## Abstract

This study aimed to investigate the correlation between instrumental activities of daily living (IADL), depression, electronic device utilization (EDU) and life satisfaction in older adults and to evaluate the dual mediating effect of EDU and life satisfaction on the relationship between IADL and depression. A secondary data analysis of the 2020 Korean Elderly Survey was carried out with 9906 older adults. The dual mediating effect was analyzed using model 6 of PROCESS Macro for SPSS v. 4.1 (New York, NY, USA). EDU (B = −0.010, 95% CI [0.007, 0.013]) and life satisfaction (B = 0.071, 95% CI [0.064, 0.079]) each had a mediating effect on the relationship between IADL and depression in older adults, and in particular, these two variables were shown to dual mediate (B = 0.017, 95% CI [0.015, 0.019]). This study confirmed that life satisfaction can be increased and depression decreased by improving the ability to use electronic devices to combat the limitations in daily functioning due to aging. It is necessary to establish a strategy to increase EDU as a part of the intervention methods for managing depression in older adults.

## 1. Introduction

The interest in successful aging is increasing as Korea’s society ages, with over 14% of the population represented by older adults over the age of 65 [1]. Successful aging refers to a state in which the levels of physical, psychological, and social functioning and adaptation to changes in life remain high with advancing age [2]. Of these, physical function refers to the ability of older adults to perform activities necessary for daily life. The ability to perform daily life tasks can be broadly divided into basic activities of daily living (ADL) and instrumental activities of daily living (IADL), wherein the former refers to tasks that are necessary for survival and the latter to those that support independence and thriving. This ability includes skills necessary for survival, such as eating or maintaining hygiene independently, and activities that improve quality of life, such as visiting the market and managing money [3]. As people age, they experience physical and psychological deterioration, which leads to decreased activity and difficulty in carrying out daily tasks [4]. This decline in their ability to perform daily activities isolates older adults, leading to psychological atrophy and depression [5]. Arguably, such depression can be a risk factor for successful aging.

Depression is the most common psychological problem in older adults, with 13.5% reporting symptoms of depression in the Korean Elderly Survey (KES) conducted in 2020 [6]. It is a mental disorder that needs appropriate management, because it reduces life satisfaction and quality of life [7] and, in severe cases, leads to suicide [8]. In recognition of its adverse impact, various studies have investigated reducing depression in older adults. One of the variables closely related to depression is life satisfaction. Since life satisfaction in older adults points to a positive cognitive judgment aspect, and depression represents a negative emotional state, it is necessary to consider the two concepts together in an empirical study [7]. Previous studies have found a negative correlation between depression and life satisfaction in older adults, that is, the higher the life satisfaction, the lower the depression [9,10]. Additionally, deterioration in physical function decreases activity levels and thereby reduces life satisfaction [11]. Considering these factors, it is necessary to examine the role of life satisfaction in the relationship between IADL and depression in geriatric populations.

Meanwhile, the ability to operate electronic devices such as smartphones and tablets or personal computers has recently been attracting attention as a variable influencing life satisfaction and depression among older adults. Due to the spread of the Internet, coupled with the developments in information technology, it has led to a rapid proliferation of digital environments in recent years [12]. With the digitization of several functions, such as searching for necessary information, maintaining contact with family and friends, or accessing necessary services, the ability to use electronic devices has a great impact on the daily life and quality of life in older adults [13]. Previous studies have found that social support and satisfaction with leisure activities influence depression in older adults [14]. The recent COVID-19 pandemic situation has brought about many limitations to the lives of older adults [15]. In such a changed environment, older adults who have a basic awareness of or education in technology can limit social interactions in their daily lives using the Internet or smartphones [15]. They can use electronic devices in place of outside activities to relieve boredom, get information on various topics, find pleasure in watching videos, and other such activities [15]. In addition, they virtually contact relatives and acquaintances using electronic devices and even participate in church services and masses held at cathedrals [15]. As such, with the increasing popularity of the Internet and smartphones, the possibility of using electronic devices as an intervention to overcome the physical limitations of older adults and improve their social health is increasing [16]. Additionally, access to digital information using electronic devices among older adults in Korea was found to have an effect on depression [12], and smartphone use affected not only depression, but also life satisfaction [17].

Therefore, it was hypothesized that (a) Electronic Device Utilization (EDU) has a mediating effect on the relationship between IADL and depression, (b) Life satisfaction has a mediating effect on the relationship between IADL and depression, and (c) Life satisfaction increased by EDU has a mediating effect on the relationship between IADL and depression. However, at present, there is insufficient supporting evidence for the Korean context. This study intends to investigate the mediating effects of EDU and life satisfaction on the relationship between IADL and depression by using data from the KES, a big data set collected from older adults in Korea. Furthermore, the results obtained could provide fundamental data for the development of interventions for depression in older adults.

## 2. Materials and Methods

### 2.1. Study Design and Participants

A secondary analysis was conducted to investigate the dual mediating effect of EDU and life satisfaction on the relationship between IADL and depression in older adults using data from the 2020 KES (https://mdis.kostat.go.kr/dwnlSvc/ofrSurvSearch.do?curMenuNo=UI_POR_P9240 (accessed on 10 July 2022). After receiving approval from the Institutional Review Board (IRB) of the Korea Institute for Health and Social Affairs, KES conducted a survey on individuals 65 years of age or older (No. 2020-36). Data collection was through one-on-one in-person interviews from 14 September to 20 November 2020. Of the 10,097 sets of data collected, a total of 9906 were analyzed, excluding samples with indirect responses or missing values.

### 2.2. Study Variables

IADL were measured using the Korean instrumental activities of daily living (K-IADL) adapted by Won et al. [3] from the tools developed by Lawton and Brody [18]. The K-IADL has a total of 10 items (e.g., items inquiring about grooming, laundry, and money management) assessing whether the respondents sought help from others for their daily activities over the past week. The total score ranges from 1 point for complete independence to 4 points for complete dependence. In this study, the total score of the K-IADL was used, and the higher the score, the higher the dependency in daily life. At the time of development of the Korean version [3], Cronbach’s ⍺ was found to be 0.83, and in this study, Cronbach’s α = 0.95

Depression was measured using the Korean short version of the Geriatric Depression Scale (SGDS-K), which was developed by Yesavage et al. [19], translated into Korean and validated by Kee [20]. It is a dichotomous scale with a total of 15 items (e.g., “Are you generally satisfied with your current life?”) asking about their mood over the past week. Reversed questions were coded in reverse, and the total score ranged from 0 to 15 points, with higher scores indicating higher levels of depressive symptoms. At the time of development of the Korean version of the tool [13], Cronbach’s α = 0.88, and in this study, Cronbach’s α = 0.85.

EDU refers to the extent of activities that an individual can perform of those possible on a personal computer, mobile phone, or tablet [21]. It also refers to the ability to utilize digital information obtained from electronic devices [21]. In this study, EDU was measured as a part of the 2020 KES and included the following 12 items: receiving messages, sending messages, information search and inquiry, taking photos or videos, listening to music, playing games, watching videos, social network services, e-commerce, financial, application search and installation, and others. If the electronic device function corresponding to each item was used, 1 point was coded, and 0 points were coded if not utilized. The score ranges from 0 to 12, with a higher score indicating greater utilization of electric devices.

Life satisfaction refers to the degree to which an older adult positively evaluates their quality of life. The scale used includes a total of seven items, including those inquiring about one’s health and economic status; relationships with spouse, children, friends, and community; social leisure and cultural activities; and overall contentment with life. It is measured on a scale of 1 point for “not at all satisfied” to 5 points for “very satisfied,” and the total score ranges from 7 to 35 points. Higher scores indicate greater satisfaction with life.

### 2.3. Statistical Analysis

The data were analyzed using IBM SPSS Statistics for Windows, Version 24.0 (IBM Corp, Armonk, NY, USA) and PROCESS Macro for SPSS v.4.1. The sample characteristics, including the study variables, were analyzed using descriptive statistics. The correlations between IADL, depression, EDU, and life satisfaction were determined through Pearson’s correlation coefficient. The dual mediating effect of EDU and life satisfaction on the relationship between IADL and depression was analyzed by applying model 6 of PROCESS Macro for SPSS v.4.1. For the significance test, a 95% confidence interval (CI) was used, and the significance level was set to 0.05.

## 3. Results

### 3.1. General Characteristics

The ages of the participants ranged from 65 to 99 years, with an average age of 73.68 years. Women were the majority at 59.9%; the sample had an average of 8.45 years of education and 96.9% literacy. Those with a spouse accounted for 59.0%, and those with children living with them accounted for only 16.0%. As for subjective health status, 49.8% answered that it was good, with 11.0% reporting a habit of smoking and 94.8% of drinking alcohol. Regular exercise was practiced by 52.3% (Table 1).

### 3.2. Descriptive Statistics of Study Variables

The average score for IADL was 10.66 (±2.71), and that for depression was 3.47 (±3.36). The average EDU score was 3.33 (±3.18) points, and the average life satisfaction score was 23.22 (±4.67) points. The most used EDU was sending a message, with 70.0%, and the least was e-commerce at only 6.6%. No-one was using electronic devices for any other purpose. (Table 2).

### 3.3. Correlation between Study Variables

Increasing dependence on others for IADL was positively correlated with depression (r = 0.34, *p* < 0.001) and negatively correlated with EDU (r = −0.17, *p* < 0.001) and life satisfaction (r = −0.28, *p* < 0.001). Depression had a negative correlation with EDU (r = −0.24, *p* < 0.001) and life satisfaction (r = −0.45, *p* < 0.001). There was a positive correlation between EDU and life satisfaction (r = 0.37, *p* < 0.001) (Table 3).

### 3.4. Mediating Effect of EDU and Life Satisfaction on the Relationship between IADL and Depression

**Figure 1:**
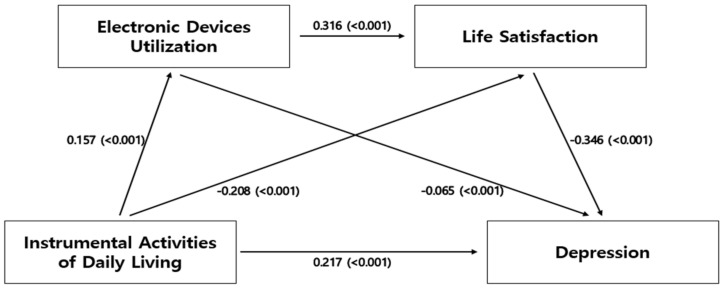
Mediating effect of variables.

In the analyses of the mediating effects of EDU and life satisfaction on the relationship between IADL and depression, each model was found to be statistically significant (Table 4, Figure 1). In Step 1, IADL had a significant effect on depression (β = 0.315, *p* < 0.001), and in Step 2, IADL had a significant effect on EDU (β = 0.157, *p* < 0.001). In Step 3, IADL (β = −0.208, *p* < 0.001) and EDU (β = 0.316, *p* < 0.001) had significant effects on life satisfaction. Furthermore, in Step 4, IADL (β = 0.217, *p* < 0.001), EDU (β = −0.065, *p* < 0.001), and life satisfaction (β = −0.346, *p* < 0.001) had significant effects on the participants’ depression.

Table 5 shows the comparison of differences in the effect values for each pathway in order to understand the significance and magnitude of the dual mediation effect. The mediating effect of IADL on depression through EDU (Indirect 1) was statistically significant (B = −0.010, 95% CI [0.007, 0.013]), and the mediating effect of IADL on depression through life satisfaction (Indirect 2) was also statistically significant (B = 0.071, 95% CI [0.064, 0.079]). Finally, the mediating effect of IADL on depression through the dual parameters of EDU and life satisfaction (Indirect 3) was also statistically significant (B = 0.017, 95% CI [0.015, 0.019]). Thus, each indirect effect significantly differed from the other. In the impact of IADL on depression, it was found that the mediating effect of life satisfaction was greater than that of EDU. Additionally, the dual mediating effect of EDU and life satisfaction was greater than EDU alone. However, it was found that the mediation of only life satisfaction was more effective than the dual mediation of EDU and life satisfaction. In other words, it was confirmed that the mediating effect of life satisfaction was the strongest in the process of IADL affecting depression.

## 4. Discussion

In this study, IADL of the participants were found to be 10.66 points, which is slightly higher than the 10.15~10.25 score in Lim and Jeon’s study on older adults [22], and lower than the 13.07 points of Seo and Song’s study [23]. The depression score was 3.47, indicating relatively low levels, since this was lower than the score of 5.37 in Lee and Oh’s [24] study of chronically ill patients using the 10th Korea Welfare Panel data in 2015 and the 7.04 obtained in Seo & Song’s study [23]. The EDU score was determined as 3.33 out of 11, and the life satisfaction was 23.22. The EDU score is similar to of the 0 to 3 out of 9 observed by Koo and Joo [21]. Life satisfaction was slightly higher than the 3.76 out of 7 reported in Ko and Moon [24] and slightly lower than the 3.65 out of 5 from Kim, Park, and Song’s study [25]. These variations could be because of the different participant characteristics in each study. In the study of Lim and Jeon [22], the age range of the participants was between 65–80 years, whereas in that of Seo and Song [23], participants 85 years or older accounted for 13.2%. Although the age range of participants in the current study was 65–99 years, the average age was 73.68 years, indicating that a majority of the sample were relatively young as compared to the aforementioned studies. In addition, in Seo and Song’s study [23], the data collection was limited to certain urban and rural areas; in Lee and Oh’s study [26], older adults with chronic diseases were targeted, and Kim et al. [25] considered only some regions in Korea. On the other hand, it can be said that the data used in this study are more reflective of the level of IADL, depression, EDU, and life satisfaction of older adults in Korea, since a large sample without age and regional bias were considered. However, while most of the variables chosen have been previously studied with the older adult population, in the case of EDU, there was no standardized tool, making it difficult to compare with other research. As the ability to utilize electronic devices and digital information becomes more important, it is necessary to develop a tool that can measure this ability in older adults.

In this study, IADL were found to have a positive direct effect on depression. In other words, the higher the dependence on IADL, the higher the depression. This mirrors the results of many previous studies [5]. IADL in older adults has been shown to affect EDU, and increased EDU has been shown to reduce depression. This result is consistent with Eom et al. [27] who found that the ability to use electronic devices in older retired men reduced depression. In addition, Park and Chung [12] reported that older adults’ access to digital information improved their ability to utilize the information, thereby reducing depression. Therefore, intervention programs and policy support to improve the ability to use electronic devices are required for this population. For older adults to be able to use electronic devices, appropriate cognitive and physical abilities are required [28,29]. However, there is insufficient evidence on the factors necessary to improve the ability of older adults to utilize electronic devices. Therefore, prior to the development of these programs, further studies are needed to explore the factors influencing EDU.

This study found that IADL of older adults influenced life satisfaction and that increased life satisfaction in turn decreased depression. This indicates that the better the ability to perform tIADL, the higher their life satisfaction. The results are consistent with Kim et al. [4] and Altun & Yaxici [10], who showed that higher life satisfaction corresponds to lower depression. Decreased physical function reduces independence in daily life. For example, in a study on driving experience of older adults, participants found it difficult to react immediately as they got older, their eyesight deteriorated, and driving became difficult due to other physical problems [30]. On the other hand, they expressed that driving makes them more mobile, enables them to attend social gatherings, and improves their quality of life through travel [30]. As such, if the ability to perform IADL is maintained, life satisfaction improves, and this, in turn, may reduce depression by enhancing the perception of the quality of life. Currently, policies to support older adults with limited capacity for basic ADL are being developed in Korea, and many will reap the benefits. However, despite the many studies conducted on improving the ability of older adults to perform ADL [30,31,32,33], programs that are actually implemented are limited. Therefore, it is necessary to apply a practical program to maintain the ability of older adults to perform IADL.

Finally, looking at the significance of the dual mediated effect, it was found that IADL in older adults increased EDU or the ability to use electronic devices, which, in turn, increased life satisfaction and, thereby, decreased depression. In the current society, if older adults are able to use electronic devices, they can easily contact their children, relatives, or acquaintances [34] and access a lot of information, which makes them feel less alienated [10]. Additionally, they can purchase basic necessities and more without having to physically move. These conveniences from the use of electronic devices can improve life satisfaction in older adults and reduce depression. Therefore, a program that can improve life satisfaction using electronic devices can be very helpful in reducing depression. However, since the use of electronic devices is affected by IADL performance and cognitive functions [20], efforts should be made to maintain the abilities necessary for the use of electronic devices in older adults.

## 5. Conclusions

This study was conducted to investigate the role of EDU and life satisfaction in the relationship between IADL and depression using the 2020 KES data. As a result of this study, it was confirmed that IADL has an effect on EDU and life satisfaction, and that improved EDU and life satisfaction could contribute to reducing depression. This study is particularly meaningful in that it provides fundamental data for preparing practical measures to improve depression in older adults by using electronic devices that help in improving their daily quality of life. In order to improve and sustain the ability of older adults to utilize electronic devices in the future, it is necessary to develop an evaluation tool to measure their ability at baseline and explore influencing factors such that appropriate programs can be developed. 

## Figures and Tables

**Table 1:** General characteristics (*n* = 9906).

Characteristics	Categories	*n* (%) or M (SD)	Range
Age		73.68 (6.45)	65–99
Sex	Male	3696 (40.1)	
	Female	5937 (59.9)	
Period of education		8.45 (4.00)	0–32
Literacy	Yes	9569 (96.9)	
	No	310 (3.1)	
Having a spouse	Yes	5844 (59.0)	
	No	4062 (41.0)	
Having children living together	Yes	1585 (16.0)	
	No	8321 (84.0)	
Perceived health status	Healthy	4935 (49.8)	
	Moderate	3118 (31.5)	
	Unhealthy	1853 (18.7)	
Smoking	Yes	1089 (11.0)	
	No	8817 (89.0)	
Drinking	Yes	9386 (94.8)	
	No	520 (5.2)	
Exercise	Yes	5180 (52.3)	
	No	4826 (47.7)	

**Table 2:** Descriptive statistics of study variables (*n* = 9906).

Variables	M (SD) Yes: *n* (%)	MIN–MAX
Instrumental Activities of Daily Living	10.66 (2.71)	10–33
Depression	3.47 (3.36)	0–15
Electronic Device Utilization	3.33 (3.18)	0–11
Receive messages (text, Kakao Talk, Telegram, etc.)	6938 (70.0)	
Sending messages (text, Kakao Talk, Telegram, etc.)	5928 (59.8)	
Information search and inquiry (news, weather, etc.)	3955 (39.9)	
Take a photo or video	4160 (42.0)	
Listen to music (MP3, radio, etc.)	2222 (22.4)	
Play games	1324 (13.4)	
Watch videos (movies, TV shows, YouTube, etc.)	2874 (29.0)	
Social network services (blog, community, band, Twitter, Facebook, Instagram, etc.)	1983 (20.0)	
E-commerce (online shopping, reservation, etc.)	653 (6.6)	
Financial transactions (Internet banking, securities, etc.)	1013 (10.2)	
Search and install applications	805 (8.1)	
Others	0 (0.0)	
Life Satisfaction	23.22 (4.67)	5–35

**Table 3:** Correlation among study variables (*n* = 9906).

Variables	IADL	Depression	EDU	LS
	r (*p*)	r (*p*)	r (*p*)	r (*p*)
Depression	0.34 (<0.001)	1		
EDU	−0.17 (<0.001)	−0.24 (<0.001)	1	
LS	−0.28 (<0.001)	−0.45 (<0.001)	0.37 (<0.001)	1

IADL: Instrumental activities of daily living, EDU: Electronic device utilization, LS: Life satisfaction.

**Table 4:** Mediating effect of EDU and life satisfaction on the relationship between IADL and depression in the older adults (*n* = 9906).

No	Variables	β (Coeffect)	SE	*p*	95% CI		R^2^
					LLCI	ULCI	
1	IADL→Depression	0.315	0.012	<0.001	0.389	0.438	0.099
2	IADL→EDU	0.157	0.009	<0.001	−0.215	−0.167	0.025
3	IADL→LS	−0.208	0.016	<0.001	−0.411	−0.345	0.164
	EDU→LS	0.316	0.013	<0.001	0.444	0.498	
4	IADL→Depression	0.217	0.012	<0.001	0.260	0.307	0.229
	EDU→Depression	−0.065	0.010	<0.001	−0.089	−0.049	
	LS→Depression	−0.346	0.007	<0.001	−0.262	−0.235	

IAD = Instrumental activities of daily living, EDU = Electronic device utilization, LS = Life satisfaction, SE = Standard Errors, LLCI = Lower limit confidence interval; ULCI = Upper limit confidence interval.

**Table 5:** Validation of mediating effect (bootstrapping) (*n* = 9906).

Variables		Effect	Boot SE	95% CI	
				LLCI	ULCI
Indirect 1	IADL→EDU→Depression	0.010	0.001	0.007	0.013
Indirect 2	IADL→LS→Depression	0.071	0.003	0.064	0.079
Indirect 3	IADL→EDU→LS→Depression	0.017	0.001	0.015	0.019
Differences (ΔB)	Indirect 1–Indirect 2	−0.061	0.004	−0.070	−0.053
	Indirect 1–Indirect 3	−0.007	0.001	−0.010	−0.003
	Indirect 2–Indirect 3	0.054	0.003	0.047	0.062

IAD = Instrumental activities of daily living, EDU = Electronic device utilization, LS = Life satisfaction, SE = Standard Errors, LLCI = Lower limit confidence interval; ULCI = Upper limit confidence interval.
